# High-throughput shoot phenotyping reveals temporal growth responses to nitrogen and inorganic and organic phosphorus sources in tomato

**DOI:** 10.1093/aobpla/plad011

**Published:** 2023-03-08

**Authors:** Hue T T Ngo, Timothy R Cavagnaro, Nathaniel Jewell, Christopher J Brien, Bettina Berger, Stephanie J Watts-Williams

**Affiliations:** The Waite Research Institute and School of Agriculture, Food and Wine, The University of Adelaide, PMB 1, Glen Osmond, South Australia, Australia; Research Institute for Forest Ecology and Environment, Vietnamese Academy of Forest Sciences, Duc Thang, Tu Liem, Hanoi, Vietnam; The Waite Research Institute and School of Agriculture, Food and Wine, The University of Adelaide, PMB 1, Glen Osmond, South Australia, Australia; The Waite Research Institute and School of Agriculture, Food and Wine, The University of Adelaide, PMB 1, Glen Osmond, South Australia, Australia; Australian Plant Phenomics Facility, The Plant Accelerator, The University of Adelaide, Glen Osmond, South Australia, Australia; The Waite Research Institute and School of Agriculture, Food and Wine, The University of Adelaide, PMB 1, Glen Osmond, South Australia, Australia; Australian Plant Phenomics Facility, The Plant Accelerator, The University of Adelaide, Glen Osmond, South Australia, Australia; The Waite Research Institute and School of Agriculture, Food and Wine, The University of Adelaide, PMB 1, Glen Osmond, South Australia, Australia; Australian Plant Phenomics Facility, The Plant Accelerator, The University of Adelaide, Glen Osmond, South Australia, Australia; The Waite Research Institute and School of Agriculture, Food and Wine, The University of Adelaide, PMB 1, Glen Osmond, South Australia, Australia; The Australian Research Council Centre of Excellence in Plant Energy Biology, The University of Adelaide, Glen Osmond, South Australia, Australia

**Keywords:** Fertilizer, high-throughput phenotyping, nitrogen, organic material, phosphorus, tomato

## Abstract

The application of nitrogen (N) and phosphorus (P) fertilizers to soils is required to maintain crop yields, so the sufficient and timely delivery of nutrients to match crop demand is important in fertilizer management. We quantified temporal growth responses of tomato plants with different rates of N and P application using high-throughput shoot phenotyping. The tomato plants were grown in soil that had organic, inorganic or a combination of sources of P incorporated. Additional N was added to each pot at low and high rates, 13 days after planting. At the same rate of total P application, the inorganic P source resulted in greater shoot growth at the early time points. Later on, the plants supplied with organic or mixed P sources grew faster than those that received the inorganic P source, resulting in comparable shoot biomass in all treatments at the time of destructive harvest. The shoot phenotyping data demonstrated that readily available soil P was important for early tomato growth while available N was more important in later stages of vegetative growth. These results suggest that a fertilizer formulation of combined inorganic and organic P sources may be able to sustain rapid and great shoot growth in tomato plants, while also reducing additional N input.

## Teaser

High-throughput phenotyping was used to track real-time plant growth of tomato plants and their responses to nutrients supplied from different sources and rates of phosphorus (P). The results suggest that the combined use of P-rich chicken litter (organic matter) and inorganic P sources can be used to close the growth gap between the sole use of organic fertilizer and the use of conventional P fertilizer. Furthermore, organic P fertilizers could reduce the need for additional nitrogen fertilizer.

## Introduction

Phosphorus (P) fertilizers are applied to soils to increase crop yields (Hopkins and Hansen, 2019) as P—a major cellular and energy constituent (e.g. of nucleic acids, phospholipids)—is the nutrient that most limits crop production globally, after nitrogen (N) (Hawkesford *et al.*, 2012). Crops have evolved a number of mechanisms by which they can acquire P from soils, such as enhancing root proliferation in soil P hotspots, increasing the number and length of root hairs, forming root clusters, modifying root architecture for more efficient P uptake (Holford, 1997; Lambers *et al.*, 2008; Funayama-Noguchi *et al.*, 2015) and forming associations with arbuscular mycorrhizal fungi (Rillig *et al.*, 2016). The uptake of P as orthophosphate occurs via plant transporters on root surfaces (Magalhaes *et al.*, 2017), meaning the acquisition of P by roots is limited by the concentration of orthophosphate in soil solution and the chance for roots to encounter P in the soil (Shen *et al.*, 2011). While applying P fertilizer increases P concentration in root rhizospheres, repeated application of P fertilizers can lead to a build-up of P in the soil if not taken up by plants (Lopez-Arredondo *et al.*, 2014), which may pollute water and adversely affect aquatic systems (Scavia *et al.*, 2014) and human health (Mallin and Cahoon, 2020). The accumulation of P in soils occurs in both conventional (Bouwman *et al.*, 2017) and organic (Cooper *et al.*, 2018) farming systems. This could be because, following fertilizer application, soluble P forms are less accessible to roots due to adsorption and precipitation reactions (McLaughlin *et al.*, 2011) or the application rates of fertilizers are high (Qaswar *et al.*, 2020). Thus, applying P fertilizers to maintain crop yields while reducing unwanted environmental impacts is an important goal.

Inorganic P fertilizers are considered conventional fertilizers, and they are derived from finite phosphate rock deposits (Ashley *et al.*, 2011). As such, the use of inorganic fertilizers is becoming more expensive and less accessible, especially to growers with low incomes or limited access to supply chains (Alewell *et al.*, 2020). In contrast, organic residues such as wheat straw and poultry manures are generated in large quantities as by-products of crop and livestock production (Sarkar *et al.*, 2020). These materials contain high concentration of nutrients, including P. There is the potential for producing readily available fertilizers for farmers who do not have access to inorganic fertilizers (Powers *et al.*, 2019; Prado *et al.*, 2022). Thus, there is potential to use P-rich organic materials to reduce reliance on inorganic P fertilizer inputs and to make use of materials that might otherwise go to waste. However, organic P sources contain lower P content per fertilizer mass in comparison with inorganic P sources (Azevedo *et al.*, 2018), and the P is released more slowly to soils because of complex organic structures (Ngo *et al.*, 2022) that need to be mineralized before it they are plant-available (Bünemann, 2015; Dey *et al.*, 2019), resulting in low crop yields (Saleem *et al.*, 2017). There is evidence that the co-application of organic materials with inorganic P sources may help to supply P in a manner that improves plant performance (Mackay *et al.*, 2017a; Timsina, 2018). In addition, the use of organic P sources could also provide N input to soils, as these materials have high N to P ratios (Ashworth *et al.*, 2020; Ngo *et al.*, 2022), it is important to understand the change in growth responses of plants to different P sources in relation to available N in the soil (Sadeghpour *et al.*, 2017) so that suitable fertilizer application could be managed.

Tomato is an important horticultural crop that is widely consumed (Capobianco-Uriarte *et al.*, 2021). Fertilizer application (including of P) for tomato plants is critical to obtain high fruit yield and quality (Wang and Xing, 2017; Filho *et al.*, 2020), so there is a need to reduce the cost and environmental impact associated with tomato fertilizer inputs. Integration of inorganic and organic fertilizer sources with soil bacteria in tomato production has shown promise, with a 25% reduction in the need for the inorganic component (Chatterjee and Bandyopadhyay, 2014). But further studies are needed to understand the underlying effects of inorganic and organic P sources on tomato growth.

High-throughput image-based phenotyping systems have been used to measure daily growth responses in a non-destructive manner (Berger *et al.*, 2012; Riley *et al.*, 2019; Watts-Williams *et al.*, 2019). The system allows us to track the magnitude and timing of effects of different fertilizer sources and their contribution to nutrient supply not only based on application rate but also to match plant demand. Here, we explored temporal responses of tomato plants to three sources, and two rates, of P (organic and/or inorganic), with two rates of N, using a high-throughput shoot phenotyping system. Specifically, our aims were:

i. To assess the impact of different P sources (organic P, inorganic P, or a combination, applied at the same total P rate) on plant growth and nutrition, at high and low application rates.
ii. To quantify if plant responses to the different P sources were affected by the further addition of N fertilizer at high and low application rates.

## Materials and Methods

### Soil, nutrient treatments and tomato plants

The soil used in this experiment was a sandy loam collected from the Waite Arboretum, South Australia (S34°58ʹ01ʹʹ, E138°37ʹ46ʹʹ) which was air-dried, sieved, mixed and stored in closed containers. Prior to use, soil was mixed with dry fine sand (1:9, w/w, referred to hereafter as ‘soil’) to reduce plant-available P of the soil and to facilitate root sampling at the end of the experiment. Furthermore, arbuscular mycorrhizal (AM) inoculant of *Rhizophagus irregularis* (DAOM181602) was also mixed with the soil (1:20, w/w) to increase AM inoculation capacity to roots. Where roots form sufficient AM colonization, they have potential benefits from AM fungi in P acquisition (Smith *et al.*, 2011; Treseder, 2013). Then the soil was packed in 1.2 L free-draining pots, each containing 1.4 kg soil substrate. In an effort to re-establish the soil microbiota and to minimise potential impacts of re-wetting soil before planting (Gao *et al.*, 2020), the soil was rewetted with 70 mL reverse osmosis (RO) water (5 %, w/w) for 3 weeks at room temperature prior to applying nutrient treatments. The soil used in this experiment contained 18.79 ± 0.22 mg kg^−1^ total available N, 3.2 ± 0.42 mg kg^−1^ plant-available P, had a pH of 5.69 ± 0.04, and electrical conductivity of 31.02 ± 2.29 μS cm^−1^ as previously reported (Ngo *et al*., 2021).

The experiment involved two determinate tomato (*Solanum lycopersicum* L.) genotypes that contrast in their ability to form AM associations, the mycorrhiza-defective mutant (*rmc:* -AM) and its wild-type progenitor (76R: +AM). Seeds of both genotypes were surface-sterilized by immersion in 70 % ethanol and 4 % NaOCl for 15 minutes, rinsed with RO water, and germinated in sand for 3 weeks to produce seedlings (first true leaf stage) before being transplanted into the prepared pots. On the day of planting (0 DAP), one tomato seedling was transplanted into each pot.

The experiment included 2×3 ×2=12 nutrient treatments, corresponding to two soil P addition rates, three P sources, and two soil N addition rates. The nutrient treatment groups were grown with two AM genotypes, making 12 × 2 = 24 treatments for the whole experiment. Phosphorus was applied to the soil at two rates: 10 (LP) and 30 (HP) mg P kg^−1^ soil as one of three P sources: P-rich organic material alone (OM-P; dry and un-ground chicken litter), inorganic P source alone (IN-P; phosphoric acid) and OM/IN-P source (1:1 mixed OM-P:IN-P ratio, mg P/mg P). The two P rates were chosen based on based on previous work using the same soil with varying levels of P applied, that showed a positive P response in 76R tomato plants when compared to no P application (Ngo *et al.*, 2021). Chicken litter used in this experiment was from the same batch that had been characterised previously (Mackay *et al.*, 2017b) and had 9.9 C/N ratio, 38.7 g N kg^−1^ and 16.5 g P kg^−1^. Chicken litter was weighed for each individual pot following corresponding P rate and P source. Inorganic P material was prepared from phosphoric acid (following Bertrand *et al.* (2006)). Specifically, 7.9 mL of 85% phosphoric acid (density = 1.685 g/cm^3^) was diluted in 500 mL reverse osmosis (RO) water that provided 42 mg P pot^−1^ in a 5 mL aliquot (equal to 30 mg P kg^−1^ soil). The diluted phosphoric acid solution was further diluted with RO water to make up 5, 10 and15 mg P kg^−1^ soil in a 5 mL volume. Phosphoric acid was used as the inorganic P source as it only supplies P nutrient to a soil with minimal effect on soil pH (Mackay *et al.*, 2017b). Nitrogen was applied as NH_4_NO_3_ solution on the soil surface of pots at two rates: 17.5 (LN) and 70 (HN) mg N kg^−1^ soil.

Phosphorus was applied to each pot immediately prior to planting by mixing potted soils with nutrient materials in plastic bags by hand for one minute, then repacked to pots. For the IN-P treatment, each pot received 5 mL pre-prepared diluted phosphoric acid, 45 mL RO water and 20 mL modified Long-Ashton mineral solution (N included and P omitted). For the OM-P treatment, each pot received pre-weighed chicken litter, 50 mL RO water and 20 mL modified Long-Ashton mineral solution. For the OM/IN treatment, each pot received 5 mL of previous prepared diluted phosphoric acid, pre-weighed chicken litter, 45 mL RO water and 20 mL of modified Long-Ashton mineral solution. All pots had 10 % moisture content (w/w) at the time of planting. Nitrogen was added to pots 13 days after planting (DAP), by dispensing either the previously prepared LN or HN solutions on soil surface before watering.

An additional set of 15 pots with LN-LP treatments were prepared in the same manner as described above with the three P sources to grow 76R tomato plants. Soil samples were taken from the additional pots on 0, 14, 28 and 42 DAP using a 10-mm diameter soil corer for plant-available P analysis (see below).

### High-throughput shoot phenotyping and plant management

The experiment was conducted in June–July (Austral winter) in a temperature-controlled greenhouse fitted with conveyor systems (NE Smarthouse) of The Plant Accelerator, Australian Plant Phenomics Facility, located at the University of Adelaide, Waite Campus, Australia (Brien *et al.*, 2013). Plants were loaded onto the conveyor system of the high-throughput phenotyping (HTP) facility at 7 DAP. With the 12 nutrient treatments, 2 genotypes and 5 replicates for each treatment combination, there was a total of n=120 pots. Each replicate occupied 2 lanes × 12 positions in the NE Smarthouse, for a total of 10 lanes × 12 positions. Randomisation was based on a latinized resolved row-column design generated using the *od* (Butler, 2018), and *dae* (Brien, 2020b) packages for the R statistical computing environment (R Core Team, 2020).

Plants were imaged daily from 7 to 42 DAP to determine shoot area using the Scanalyzer 3D imaging system (LemnaTec GmbH, Aachen, Germany) (Berger *et al.*, 2012). Red–green–blue (RGB) images were taken from three views, comprising two side views at an angular separation of 80° and a view from above. Images were captured with 8-megapixel cameras (GT3300C, Allied Vision Technologies, Germany) in an imaging cabinet with black background and LED flash lighting. Images had a resolution of 43 pixels/cm and were analysed using the LemnaGrid software package (LemnaTec GmbH, Aachen, Germany). The approach followed the process previously described (Neilson *et al.*, 2015). In brief, a nearest-neighbour colour classification was used to separate foreground (plant) from background, followed by noise reduction steps. Finally, all elements identified as plant were composed into a single object and size of the object was measured in pixels. The pixels from the three images were summed as projected shoot area (PSA) (kilopixels), which has been previously shown as a good predictor of shoot biomass for various species (Honsdorf *et al.*, 2014; Neilson *et al.*, 2015; Al-Tamimi *et al.*, 2016). Plants were watered to 10 % (w/w) gravimetric water content by the automated system, on a daily basis, a water-level sufficient for plant growth in a sandy soil mix with a low field capacity. A modified Long-Ashton mineral solution (N included and P omitted) (following Cavagnaro *et al.* (2001)) was supplied to the plants at a rate of 20 mL pot^−1^ on 6, 20, 27 and 34 DAP. During the experiment, the ambient temperature was maintained at an average of 24 °C/17 °C day/night cycle. The greenhouse used natural lighting without supplementary lighting and the average light levels at midday were 280 µmol m^−2^ s^−1^ and the average day length was nine hours, which was adequate for growing tomato plants in winter (Tartachnyk and Blanke, 2007).

### Harvesting, soil and plant analysis

At the time of planting, soil sub-samples (50 g) were taken from each pot to quantify plant-available P (Murphy and Riley, 1962) in 0.5 M NaHCO_3_ at pH 8.5, and available N in 2M KCl (sum of ammonium (Forster, 1995) and nitrate (Miranda *et al.*, 2001)). On 42 DAP, all tomato plants were destructively harvested. Shoots were cut at soil level and weighed, and then dried in an oven for a week at 60 °C and weighed again. The dry shoots were then ground to a fine powder using a puck mill pulverizer machine and analyzed for total P concentration, following digestion in concentrated nitric acid and 36% hydrogen peroxide (1:4, v/v), P concentration was measured by inductively coupled plasma optical emission spectroscopy (ICP-OES) (following Wheal *et al.* (2011)). Roots were washed with RO water to remove any soil and blotted dry. Subsamples of ~200 mg fresh roots were taken and fixed in 50 % ethanol for 24 h. Fixed roots were rinsed with RO water and then cleared in 10 % potassium hydroxide at room temperature for seven days. Cleared roots were rinsed and then stained in 5 % ink in vinegar at 60 °C for 15 min (Vierheilig *et al.*, 1998), then de-stained in acidified water for 24 h, before being stored in 50 % glycerol solution. Percent root length AM colonization was estimated on stained root samples according to the gridline intersect method at 20 × magnification (Giovannetti and Mosse, 1980).

### Data processing, calculation and statistical analysis

The data for analysis were prepared by applying the Smoothing and Extraction of Traits (SET) method (Brien *et al.*, 2020) to the imaging data, using the *growthPheno* package (Brien, 2020c) with the R statistical computing environment (R Core Team, 2020). The raw data for DAP 34 were removed from the data set because the plants were noticeably water-stressed. Spline smoothing was applied to the PSA curve of each plant to remove transient fluctuations in the trend over time, yielding smoothed projected shoot area (sPSA) (kilopixels). Data smoothed using different degrees of freedom were compared by using *probeSmoothing* from *growthPheno,* after which six degrees of freedom (df = 6, mild smoothing) was chosen subjectively as appropriate for smoothing this dataset and sPSA obtained. Then the smoothed absolute growth rate (sPSA AGR, kilopixels/day) describes the estimated daily rate of accumulation of shoot biomass and was calculated based on the sPSA data. In particular, the sPSA AGR from DAP t1 to t2 is given in Equation (1), where sPSA_t1_ and sPSA_t2_ are the projected shoot areas at t1 and t2, respectively:


$$\begin{array}{l} sPSA\;\;\;AGR=\frac{sPSA_{t2}-sPSA_{t1}}{t2-t1} \end{array}$$ (1)

To investigate the growth dynamics, the smoothed data was used to produce single-day responses sPSA at DAP 13, 16, 19, 22, 25, 30, 36 and 42; and interval responses for sPSA AGR at 13–16, 16–19, 19–22, 22–25, 25–30, 30–36 and 36–42. Maximum growth rate (sPSA AGR_Max_) and corresponding date (sPSA AGR_Max.DAP_) were computed over all imaging days.

To produce phenotypic predictions, or adjusted means considering the effects of position within the Smarthouse and treatment factors, a fixed-model analysis was performed for each imaging or harvest trait using *ASReml-R* (Butler *et al*., 2020) and *asremlPlus* (Brien, 2020a) packages with the R statistical computing environment (R Core Team, 2020). The maximal model for this analysis is of the form (Equation 2)


$$\begin{array}{l} y=X\beta +e \end{array}$$ (2)

where **y** is the response vector of values for the trait being analysed; β is the vector of fixed effects, with design matrix ***X***, **e** is the vector of residual effects. The fixed-effects vector β is partitioned as $\left[\begin{array}{l} \mu \;\beta_{xPosn}\;\beta_{B}^{⊤}\;\beta_{G}^{⊤}\;\beta_{76R}^{⊤}\;\beta_{rmc}^{⊤} \end{array}\right]$, where 𝜇 is the overall mean, β_*x*Posn_ allows for a linear east–west trend across positions within the Smarthouse and the β subvectors allow for consistent differences between Blocks (B), a consistent difference between the two Genotypes (G), and the three-way factorial effects of the nutrient factors treatment factors N rate (N), P rate (P) and P source (S) on the genotype 76R and on the genotype *rmc*. In order to investigate the effects of N, P and S within each Genotype, each of the subvectors $\beta_{76R}$ and $\beta_{rmc}$ is partitioned into subvectors as follows $\left[\begin{array}{l} \beta_{N}^{⊤}\;\beta_{P}^{⊤}\;\beta_{S}^{⊤}\;\beta_{N:P}^{⊤}\;\beta_{N:S}^{⊤}\;\beta_{P:S}^{⊤}\;\beta_{N:P:S}^{⊤}\; \end{array}\right]$, where $\beta_{N}$, $\beta_{P}$ and $\beta_{S}$ correspond to the main effects, $\beta_{N:P}$, $\beta_{N:S}$ and $\beta_{P:S}$ correspond to the two-factor interactions, and $\beta_{N:P:S}$ corresponds to the three-factor interaction of N, P and S for a Genotype. The residual effects e were assumed to be normally distributed with variance $\sigma^{2}$, except that for some traits (shoot fresh/dry weight and shoot P content), the variance was allowed to differ between combinations of N and P. All residual plots were satisfactory, indicating that the fitted model appeared to be appropriate.

In the case of the sPSA AGR_Max.DAP_ trait, Equation (2) was modified to account for the exclusion of all plants for two of the combinations of N and S as these plants had not yet achieved peak growth by the end of the imaging period. A nested model was used in which the effects of P and S were examined within the combinations of two Nitrogen levels and two Genotype levels. The vector β is now partitioned as $\left[\begin{array}{l} \mu \;\beta_{xPosn}\;\beta_{B}^{⊤}\;\beta_{N:G}^{⊤}\;\beta_{76R^{-}}^{⊤}\;\beta_{rmc^{-}}^{⊤} \end{array}\right]$, where $\beta_{N:G}$ is the subvector of the effects for the combinations of N and G and each of $\beta_{76R^{-}}^{⊤}$ and $\beta_{rmc^{-}}^{⊤}$ is partitioned as $\left[\begin{array}{l} \beta_{P*S_{LN}}^{⊤}\;\beta_{P_{HN}}^{⊤}\; \end{array}\right]$, where $\beta_{P*S_{LN}}$ is the subvector for the main effects and two-factor interactions for P and S within the low Nitrogen level (LN) for a genotype and $\beta_{P_{HN}}$ is the subvector for the main effects P within the high nitrogen level (HN) for a genotype.

For each imaging and harvest trait, Wald *F*-statistics were produced for the treatment main effects and interactions, and these were used to identify a chosen model based on the statistically significant nutrient treatment terms for each genotype. Phenotypic predictions conforming to the chosen model were then obtained for each combination of N addition rate, P addition rate and P source for the Genotype 76R. Finally, least significant differences [LSD (5%)] were calculated for comparing pairs of predictions within a trait for the Genotype 76R. The analysis focused on Genotype 76R from here onwards, since the AM colonization was very low (<10%) thus genotypic comparison was not considered appropriate.

## Results

### Phenotyping of shoot growth over time

**Figure 1.**
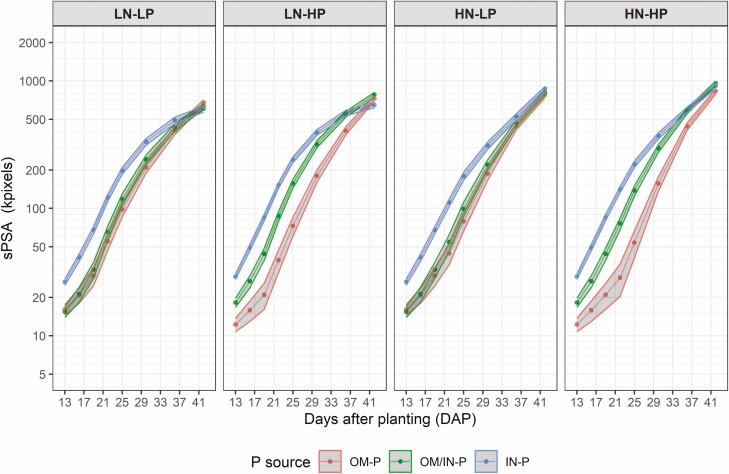
Phenotypic predictions, plotted on a log scale, for the smoothed projected shoot area (sPSA) over DAP 13–42 for tomato plants grown in three P sources (OM-P, OM-P/IN-P and IN-P) and grouped by the four combinations of N rates (LN and HN) and P rates (LP and HP). The widths of the ribbons are equal to the least significant differences within a DAP. The predictions are not significantly different (_*p*_*>* 0_._05) where the ribbons overlap.

**Figure 2.**
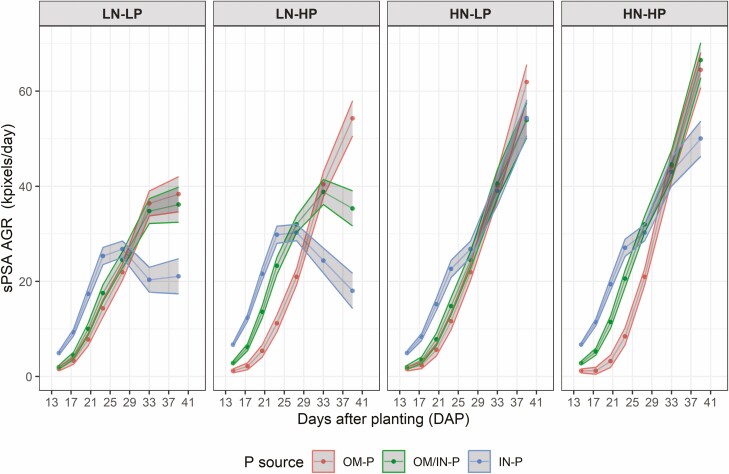
Phenotypic predictions for the smoothed absolute growth rates (sPSA AGR) over DAP 13–42 for tomato plants grown in three P sources (OM-P, OM-P/IN-P and IN-P) and grouped by the four combinations of N rates (LN and HN) and P rates (LP and HP). The widths of the ribbons iare equal to the least significant differences within a DAP. The predictions are not significantly different (*P >* 0_._05) where the ribbons overlap.

**Figure 3.**
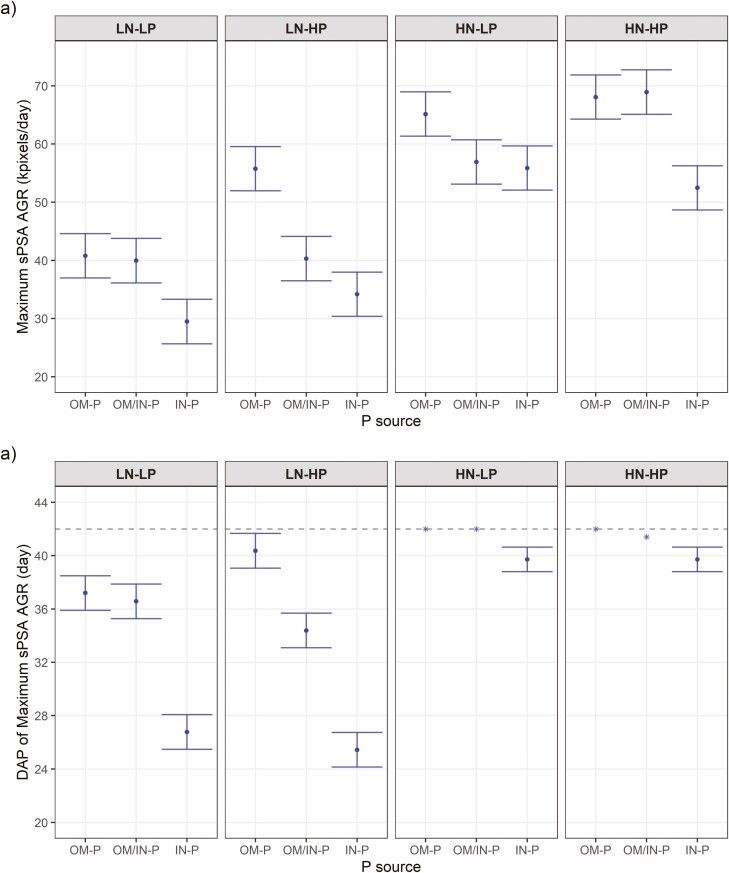
Phenotypic predictions for the maximum growth rate (a) and the corresponding DAP (b) at the destructive harvest for tomato plants grown in three P sources (OM-P, OM-P/IN-P and IN-P) and grouped by the four combinations of N rates (LN and HN) and P rates (LP and HP). Error bars are equal to a prediction _*±*_ half-LSD (5%). Error bars within an N rate that overlap indicate that the prediction are not significantly different (*P>* 0_._05). The asterisks (‘_*_’) indicate that the maximum sPSA AGR occurred at the end of imaging (DAP 41–42) for all plants without sPSA AGR having peaked and so the DAP mean is presented (the dashed line marks DAP 42).

Shoot area (as sPSA) was affected by the interaction of P source and rate prior to 36 DAP, with plants that received the IN-P source having greater sPSA than the OM/IN-P and OM-P sources and the sPSA for different P sources being more separated at HP than at LP (Fig. 1, [see Supporting information—Table S1]). Similarly, sPSA AGR of plants grown with the IN-P source had higher sPSA AGR than the plants grown with OM/IN-P and OM-P sources for the first four time intervals (13–25 DAP) (Fig. 2, [see Supporting information—Table S1]). In the last four time intervals (spanning 29–42 DAP), the sPSA AGR of the OM/IN-P and OM-P sources were similar or higher than that of the IN-P source, leading to greater sPSA AGR_max_ value in the sources containing organic P compared to the inorganic P source, despite taking longer to reach maxium growth rate (Fig. 3).

### Effect of P source and rate on soil nutrients, plant biomass and nutrition at harvest

At the time of planting, available P of the soils was higher in IN-P source than in OM-P sources, and intermediate in OM/IN-P source in low P application rate. Despite that, available N of the low P soils was not different among different P sources (Table 1). At the high P application rate, whereas the pattern of available soil P was similar among different P sources, available N was highest in OM-P source.

**Table 1.** Characteristics of the soil at the time of planting after incorporating three P sources: OM-P, OM/IN-P and IN-P. Values are mean ± SEM, *n* = 10. Within column, means followed by different letters are significantly different at the P < 0.05 level.

P rate	P source	Plant-available P (mg kg^-1^) Day 0	Available N (mg kg^-1^)
**LP**	IN-P	18.94 ± 0.73(ab)	27.57 ± 0.56(b)
	OM/IN-P	9.24 ± 2.37(bc)	29.38 ± 0.76(b)
	OM-P	3.50 ± 1.54(c)	31.07 ± 0.63(b)
**HP**	IN-P	27.77 ± 2.47(a)	27.62 ± 0.88(b)
	OM/IN-P	18.15 ± 2.19(ab)	36.36 ± 1.77(b)
	OM-P	20.73 ± 5.68(ab)	65.68 ± 4.04(a)

**Figure 4.**
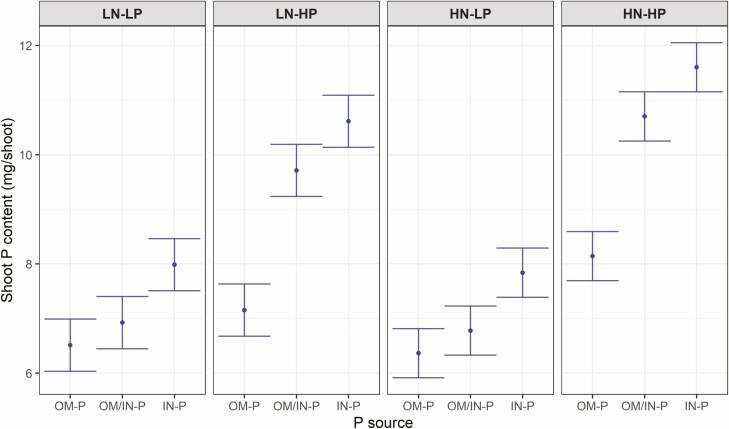
Phenotypic predictions for Shoot P content at the destructive harvest for tomato plants grown in three P sources (OM-P, OM-P/IN-P and IN-P) and grouped by the four combinations of N rates (LN and HN) and P rates (LP and HP). Error bars are equal to a prediction _*±*_ half-LSD (5%). Error bars within N rate that overlap indicate that the predictions are not significantly different (*P* > 0.05).

**Figure 5.**
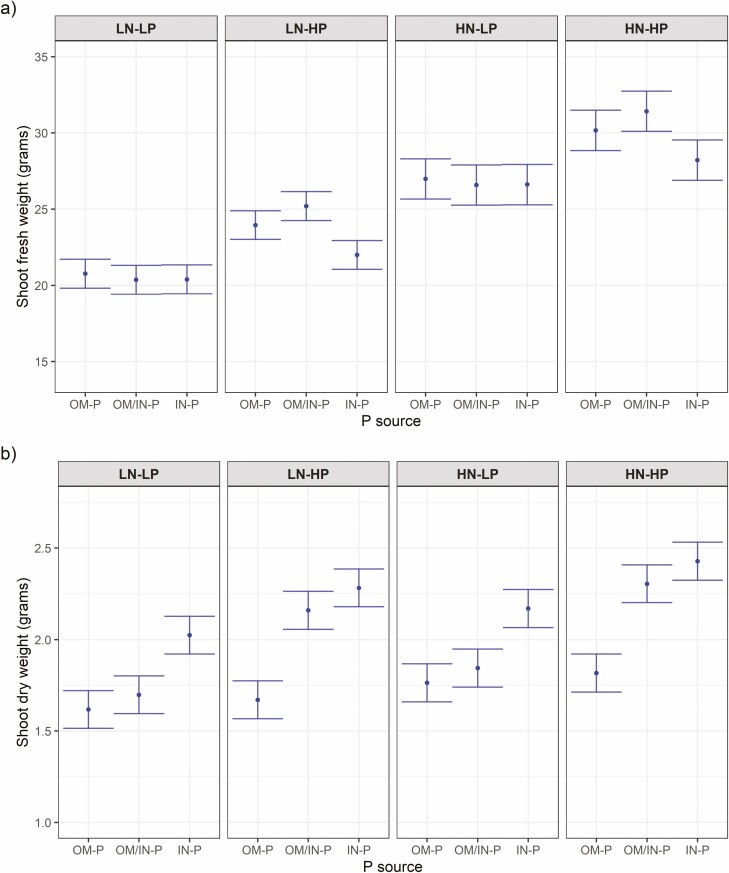
Phenotypic predictions for Shoot fresh weight (a) and Shoot dry weight (b) at the destructive harvest for tomato plants grown in three P sources (OM-P, OM-P/IN-P and IN-P) and grouped by the four combinations of N rates (LN and HN) and P rates (LP and HP). Error bars are equal to a prediction _*±*_ half-LSD (5%). Error bars within N rate that overlap indicate that the predictions are not significantly different (*P* > 0.05).

At the time of harvest, shoot P content showed the same trend as initial plant-available soil P where the differences in shoot P contents among three P sources separated more at the HP than the LP rate (Fig. 4). Shoot fresh weight (SFW) and shoot dry weight (SDW) were affected by the interaction of P source and P rate (Fig. 5, [see Supporting information—Table S1]). SFW were similar among different P sources at the low P rate, whereas SFW was higher in OM/IN-P source compared to the IN-P sources at the high P rate. The pattern of SFW was correlated linearly with the final day sPSA data ([see Supporting information—Fig. S1]).

### Effect of N rate on shoot growth response over time

Nitrogen did not have main or interacting effects on sPSA and sPSA AGR prior to 19 DAP ([see Supporting information—Table S1). At 36 DAP, while high N increased sPSA and sPSA AGR compared to low N, the N rate did not interact with the different P sources to affect shoot growth. However, from 36 DAP onwards, the sPSA and sPSA AGR were affected by the interaction of N rate and P source and/or P rate. Specifically, later sPSA and sPSA AGR values of plants supplied with OM-P were similar or higher than other P sources, regardless N and P application rates.

## Discussion

The P source and rate had an interactive effect on shoot growth response over the course of the experiment. The IN-P source produced greater shoot growth compared to the other P sources in the early growth stages (prior 25 DAP), while the shoot growth rates for the different treatments diverged later (from 25 DAP onward) as N rate became the main or the interactive statistical effect. The use of high-throughput shoot phenotyping highlights the complex and temporally dynamic responses of plants to the form of soil P fertilizer, as well as P and N application rates, and help to elucidate plant nutrient demands over time.

### Timing of shoot growth reflected the dynamic nature of soil P availability

The nature of the source of supplied P (organic, inorganic or a combination) had a large impact on the amount of plant-available P in the soil. Here, the organic P source provided up to a half of plant-available P compared to the inorganic P source, despite their being applied to the soil in equal total P amounts. This agrees with previous research where organic P sources supplied less rapidly-available P to plants compared to an inorganic P source (Ngo *et al.*, 2022). This was not unexpected given that only 37 % of total P in the chicken litter used here is present in a plant-available form (Mackay *et al.*, 2017b). Over time, we also reported a decreasing trend of plant available soil P of the three P sources with the remaining trend of highest available P in the inorganic P source ([see Supporting information—Fig. S2]).

Subsequently, the larger discrepancies in shoot growth responses observed between different P sources was most likely due to the large difference of initial available soil P. In addition, available soil P also affected plant P uptake as analysed in the shoot tissues at harvest, where plants grown with the organic P source did not accumulate as much P in the shoots as those supplied with the inorganic P source, consistent with previous research (Mackay *et al.*, 2017b). On the other hand, the combined use of inorganic and organic sources of P resulted in the same, or better, shoot growth (both over time and at harvest) as compared to the organic P source alone; this is likely due to inorganic P source supplying P early, and organic P source supplying P to the plants in later stages of development. In addition, the combined use of inorganic and organic sources of P possibly reduced the C:P ratio of the amendment, which in turn may have enhanced microbial P mineralization and plant P uptake (Zhang *et al.*, 2014). Thus, the evidence of positive shoot growth responses to the combined P source provides a case for using the combined sources of P as fertilizer to reduce mined P resources as well as recycle agricultural wastes.

### The additional N in the organic P source aided plant growth

The application and availability of N were highly interactive with P fertilizer in determining the timing and magnitude of shoot growth. Shoot growth rate was stimulated by inorganic P in the early stages, but growth rate was quickly restricted later, unless the high N treatment was applied. The reduction in growth can be explained by the lower available N status of the soil in the inorganic P source as compared to the OM-P and the mixed P sources. These results demonstrate a benefit of the organic P source in aiding plant N nutrition and thus sustaining plant growth. In addition, the growth rates of the plants supplied with organic P sources were as high as that of inorganic P sources with high N and P applications, indicating that the fate of N nutrient release from organic P sources could match the plant N demand over its life. This agrees with previous research where using combined inorganic fertilizer and organic materials reduced the demand on inorganic fertilizer by up to 50% without a yield reduction (Hernández *et al.*, 2014; Moe *et al.*, 2019). In addition, the use of organic materials as N and P fertilizers may help to mitigate the risk of N and P losses via leaching (Li *et al.*, 2012; Soto *et al.*, 2015). This result highlights that organic P sources such as chicken litter may have indirect benefits such as contributing N to sustain plant growth, thereby reducing the dependence on conventional sources of N fertilizer.

## Conclusions

We used a high-throughput phenotyping system to investigate tomato shoot growth over time, in plants fertilised with different P sources and rates, and with further application of low and high N rates. The inorganic P source alone led to rapid early shoot growth compared to the P-rich organic source alone, or the combination of the two sources, which was likely due to more readily-available P of the inorganic P source. Further N addition improved plant growth responses in later stage due to later N exhaustion. However, the original N from organic P source reduced the need for additional N (as ammonium nitrate) fertilizer. Taken together, the results suggest that the combined use of P-rich organic materials and inorganic P sources can be used to close the growth gap between organic and inorganic P sources, and that organic P sources could also reduce the need for additional N fertilizer.

## Supplementary Material

plad011_suppl_Supplementary_Material

## Data Availability

Data sharing is not applicable to this article as all created data is already contained within this article or in the supplementary material.
