# Supplementary material for: High-throughput shoot phenotyping reveals temporal growth responses to nitrogen and inorganic and organic phosphorus sources in tomato
Source: AoB Plants. 2023 Mar 8;15(2):plad011. doi: 10.1093/aobpla/plad011 (PMC10129286; doi:10.1093/aobpla/plad011)
Supplement: plad011_suppl_Supplementary_Material [file plad011_suppl_supplementary_material.docx]

**Supplementary information**

Table 1: The *_p_*-values for the Wald F-statistics of 2^3^-1=7 treatment main effects and interactions for the factors nitrogen application rate (N), phosphorus application rate (P) and phosphorus source (S); the *_p_*-values for sPSA AGR Max DAP are not given, being derived using a unique model. The last column has the model chosen for each trait using these *_p_*-values. Statistically significant terms (*_p ≤_* 0*_._*05) are flagged with an asterisk (*). Wherever an interaction term is significant, the corresponding lower-level p-values are marked as not applicable (na).

| Trait | N:P:S | N:P | N:S | P:S | N | P | S | Chosen model |
| --- | --- | --- | --- | --- | --- | --- | --- | --- |
| sPSA 13 | 0.695 | 0.081 | 0.216 | * 0.011 | 0.268 | na | na | P:S |
| sPSA 16 | 0.863 | 0.233 | 0.239 | * 0.002 | 0.376 | na | na | P:S |
| sPSA 19 | 0.941 | 0.642 | 0.202 | * <0.001 | 0.106 | na | na | P:S |
| sPSA 22 | 0.875 | 0.966 | 0.127 | * <0.001 | * 0.020 | na | na | P:S + N |
| sPSA 25 | 0.844 | 0.849 | 0.114 | * <0.001 | * 0.010 | na | na | P:S + N |
| sPSA 30 | 0.725 | 0.889 | 0.378 | * <0.001 | * 0.041 | na | na | P:S + N |
| sPSA 36 | 0.499 | 0.369 | 0.131 | * 0.005 | * 0.021 | na | na | P:S + N |
| sPSA 42 | 0.052 | 0.521 | * 0.002 | * 0.042 | na | na | na | P:S + N:S |
| sPSA AGR 13-16 | 0.976 | 0.885 | 0.405 | * <0.001 | 0.691 | na | na | P:S |
| sPSA AGR 16-19 | 0.698 | 0.594 | 0.163 | * <0.001 | * 0.023 | na | na | P:S + N |
| sPSA AGR 19-22 | 0.711 | 0.520 | 0.081 | * <0.001 | * 0.003 | na | na | P:S + N |
| sPSA AGR 22-25 | 0.741 | 0.687 | 0.136 | * <0.001 | * 0.006 | na | na | P:S + N |
| sPSA AGR 25-30 | 0.436 | 0.549 | 0.769 | * 0.002 | 0.466 | na | na | P:S |
| sPSA AGR 30-36 | 0.061 | 0.116 | * <0.001 | 0.250 | na | * 0.001 | na | N:S + P |
| sPSA AGR 36-42 | * 0.002 | na | na | na | na | na | na | N:P:S |
| sPSA AGR Max | * 0.006 | na | na | na | na | na | na | N:P:S |
| sPSA AGR Max DAP | na | na | na | na | na | na | na | P:S[LN] |
| Shoot FW | 0.387 | 0.824 | 0.771 | * 0.026 | * <0.001 | na | na | P:S + N |
| Shoot DW | 0.765 | 0.883 | 0.811 | * 0.014 | * 0.010 | na | na | P:S + N |
| Shoot P content | 0.124 | * 0.025 | 0.889 | * <0.001 | na | na | na | P:S + N:P |


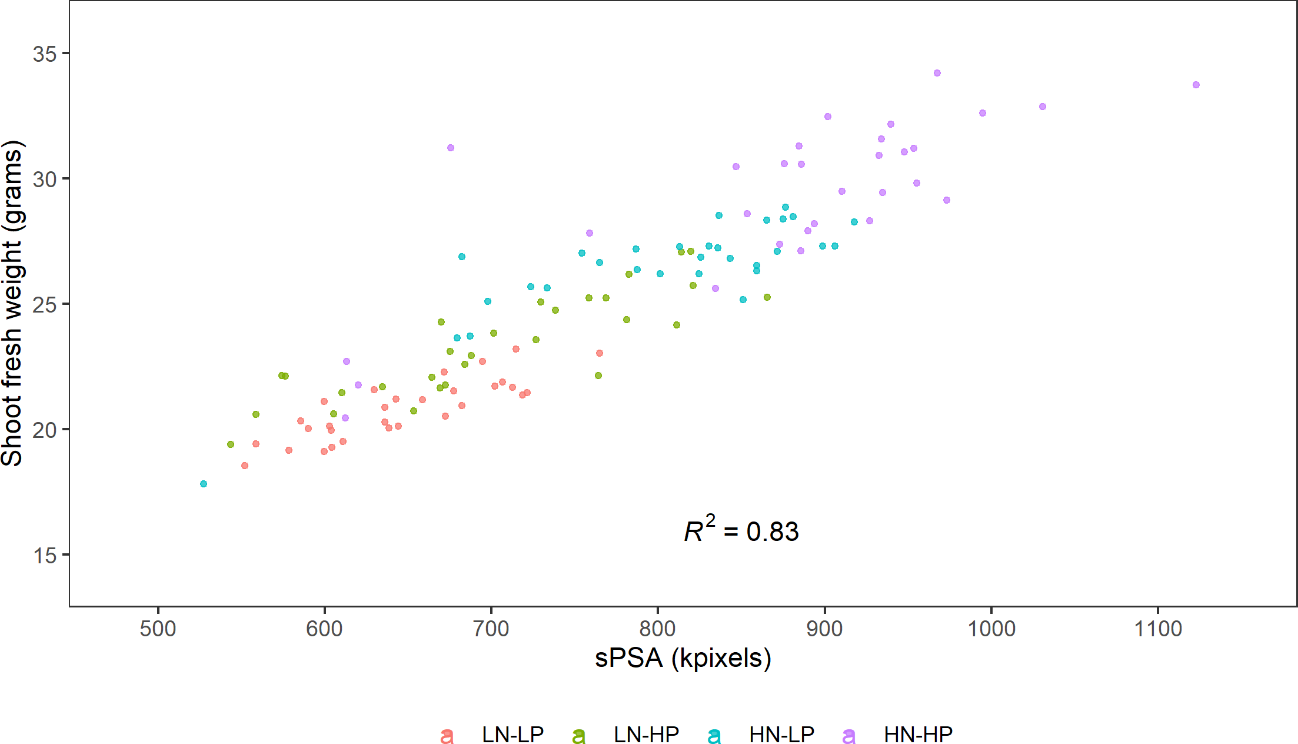


Figure S1. Linear relationship between shoot fresh weight and final smoothed projected shoot area (sPSA) (DAP 42).

Figure S2. Change in plant-available P over time in soil grown the tomato genotype 76R at 10 mg kg^-1^ total P provided from one of three P sources: all inorganic P (IN-P), a blend of inorganic and organic P (OM/IN-P), or all organic P (as chicken litter) (OM-P).
